# Gaussian Process Regression Tuned by Bayesian Optimization for Seawater Intrusion Prediction

**DOI:** 10.1155/2019/2859429

**Published:** 2019-01-17

**Authors:** George Kopsiaftis, Eftychios Protopapadakis, Athanasios Voulodimos, Nikolaos Doulamis, Aristotelis Mantoglou

**Affiliations:** ^1^National Technical University of Athens, 15773 Athens, Greece; ^2^Department of Informatics and Computer Engineering, University of West Attica, 12243 Athens, Greece; ^3^Institute of Communication and Computer Systems (ICCS), Zografou 15773, Athens, Greece

## Abstract

Accurate prediction of the seawater intrusion extent is necessary for many applications, such as groundwater management or protection of coastal aquifers from water quality deterioration. However, most applications require a large number of simulations usually at the expense of prediction accuracy. In this study, the Gaussian process regression method is investigated as a potential surrogate model for the computationally expensive variable density model. Gaussian process regression is a nonparametric kernel-based probabilistic model able to handle complex relations between input and output. In this study, the extent of seawater intrusion is represented by the location of the 0.5 kg/m^3^ iso-chlore at the bottom of the aquifer (seawater intrusion toe). The initial position of the toe, expressed as the distance of the specific line from a number of observation points across the coastline, along with the pumping rates are the surrogate model inputs, whereas the final position of the toe constitutes the output variable set. The training sample of the surrogate model consists of 4000 variable density simulations, which differ not only in the pumping rate pattern but also in the initial concentration distribution. The Latin hypercube sampling method is used to obtain the pumping rate patterns. For comparison purposes, a number of widely used regression methods are employed, specifically regression trees and Support Vector Machine regression (linear and nonlinear). A Bayesian optimization method is applied to all the regressors, to maximize their efficiency in the prediction of seawater intrusion. The final results indicate that the Gaussian process regression method, albeit more time consuming, proved to be more efficient in terms of the mean absolute error (MAE), the root mean square error (RMSE), and the coefficient of determination (*R*^2^).

## 1. Introduction

Seawater intrusion (SI) in coastal aquifers is a complex physical phenomenon, consisting of several physical processes. A number of approaches have been proposed to simulate SI, considering different components. Dispersion mechanisms and water density changes are considered critical components in the accurate representation of SI [1]. Both mechanisms are incorporated in the mathematical description of what is known as variable density (VD) models. Although accurate, VD models are CPU intensive and entail long runtimes because the resulting model equations are solved using complex numerical methods (e.g., finite differences and finite element methods). The time-consuming simulations hinder the exploitation of the high accuracy VD models in applications which require a large number of iterations, such as coastal groundwater management, parameter estimation, sensitivity analysis, and uncertainty analysis. Because of the long runtimes, it is also rather impractical to incorporate VD models in real-time systems, e.g., decision support systems [2]. A common method to tackle the duration problem is the use of very fast approximation models, which could efficiently substitute the original VD models, without compromising the accuracy of the results. These models are usually called surrogate models, metamodels, model emulators, lower fidelity models, proxy models, and response surfaces [2–5].

Surrogate model practice is based on the notion that original model response(s) could be approximated by a computationally more efficient model, for a range of values of the selected model variables [5]. In the present study, the Gaussian process regression (GPR) as a surrogate model for SI is examined. Rajabi and Ketabchi [6] summarized the advantages of GPRs compared with other surrogate models in the following: (i) GPRs provide both an approximation of the original high-fidelity model results and a probabilistic estimate of the approximation uncertainties [7, 8], (ii) GPRs' structure is relatively simple based on the mean and covariance functions [9], (iii) GPRs are flexible with regard to the probability distributions of the input data, (iv) GPRs can efficiently cope with models of different complexity [10, 11], (v) GPRs provide the ability to calculate the mean and standard deviation, and (vi) GPRs provide the ability to incorporate prior knowledge of the outputs in the metamodel construction process [12].

The GPR results are compared with other widely used methods, specifically, linear regression (LR), support vector machine regression (SVMR), binary regression decision tree (BRDT), and ensemble tree learners (ETL). It should be noted that the examined methods are all univariate. A Bayesian optimization is employed in all surrogate models, to improve their efficiency.

The remainder of this study is structured as follows: Section 2 presents a brief survey of the related work. In Section 3, the seawater intrusion model is described, whereas section 4 presents the proposed Gaussian process regression scheme and the Bayesian optimization process. In Section 5, the experimental evaluation is provided, and finally, section 6 concludes the paper.

## 2. Related Work

Approximation models have been widely used during the last decade in water resources (e.g., [13, 14]) and especially in groundwater modelling. Razavi et al. [5] and Asher et al. [2] performed an extended review of surrogate model applications in water resources field. Regarding coastal aquifers, surrogate models have been widely used for the prediction of SI, substituting the complex fluid flow and transport processes. For example, Bhattacharjya et al. [15] employed artificial neural networks (ANN) to approximate density-dependent flow in coastal aquifers. In more recent studies, Roy and Datta [16] used the fuzzy C-mean clustering method to predict SI, while Lal and Datta [17] investigated the ability of Support Vector Machine regression (SVMr) to predict the location of SI toe and concluded that the method surpasses other widely used metamodelling methods, such as genetic programming (GP).

A significant number of relative studies are devoted to the use of surrogate models in coastal aquifer management problems to cope with the computational burden, which arises from simulation-optimization schemes [18]. A well-established metamodel which is very common in coastal aquifer management literature is the artificial neural networks (ANNs) [5, 18]. Bhattacharjya and Datta [19] employed an ANN model to approximate a density-dependent model in a genetic optimization framework. Rao et al. [20] and Kourakos and Mantoglou [21] incorporated ANNs in a simulation-optimization scheme to replace the SEAWAT numerical code. Kourakos and Mantoglou [22] proposed a pumping optimization method based on modular neural networks and an Evolutionary Annealing Simplex optimization algorithm. Ataie-Ashtiani et al. [23] combined a simulation-optimization procedure with ANNs to develop an efficient model for the multiobjective management of groundwater lenses in small islands. Christelis and Mantoglou [24] used cubic radial basis functions (RBFs) in two adaptive metamodeling frameworks: (1) the adaptive-recursive approach and (2) the metamodel-embedded evolutionary strategy. The latter proved to be computationally more efficient, providing solutions near the global optimum. Christelis et al. [25] employed two surrogate-based optimization (SBO) frameworks, under restricted computational budgets to improve the efficiency of optimization algorithms in problems of moderate and large dimensionalities. In a more recent study, Christelis and Mantoglou employed variable-fidelity surrogate models and evolutionary algorithms to calculate the maximum allowed pumping rates in coastal aquifers. Sreekanth and Datta in several studies [26–28] examined genetic programming (GP) as a potential surrogate model in multiobjective management of SI in coastal aquifers and compared the proposed method with modular neural network metamodels. Roy and Datta examined several surrogate models to predict SI in coastal aquifers and employed all these models in coastal aquifer management problems [29–31]. A review of surrogate models, focusing on SI and coastal aquifer management, is presented by Roy and Datta [32].

Gaussian process metamodels have been widely used in engineering optimization applications, but according to Razavi et al. [5] and Asher et al. [2], they have not yet attracted much attention in groundwater field, particularly SI. Stone [33] presented a Bayesian emulation methodology as an alternative to Monte Carlo in the analysis of stochastic groundwater models. Zhang et al. [34] employed an adaptive Gaussian process-based method to identify contaminant source in groundwater problems, whereas Crevillén-García et al. [35] used Gaussian process method to perform uncertainty analysis in a convectively enhanced dissolution process model. Raghavendra and Deka [36] used GPR and adaptive neuro fuzzy inference system (ANFIS) to forecast groundwater level time series. In a recent study, Rajabi and Ketabchi [6] used Gaussian process emulators in a simulation-optimization framework to address the computational challenges arising from the large number of the required simulations. Roy and Datta [37] incorporated three metamodels, particularly ANFIS, GPR, and multivariate adaptive regression spline (MARS), in a multiobjective optimization framework to quantify the influence of sea-level rise on coastal aquifer management. In this specific study, they concluded that the ANFIS-based metamodel proved to be more efficient and inexpensive compared with the other two metamodels. The authors also performed a comparative analysis between several surrogate models [38], including GPR, in a coupled simulation-optimization methodology under parameter uncertainty. In this specific study, they concluded that the GPR metamodels and their ensemble (EGPR) proved to be more efficient in terms of prediction compared with other similar methods, such as the MARS metamodel and the regression tree (RT) metamodel.

## 3. Seawater Intrusion Model

### 3.1. Variable Density and Salt Transport Model

As mentioned in section 1, VD models are based on the spatial variability of groundwater density, which ranges from saline water density to freshwater density. The driving force of the seawater/freshwater mixing is the dispersion mechanism, which results in the existence of a transition zone across the entire coastline. The width and exact position of the zone depends on the aquifer parameters and the pumping regime. In the current study, thermal and viscosity effects are neglected and the density changes are attributed only to concentration effect. The flow and solute transport equations are used to describe mathematically the VD model. The two equations form a coupled differential equation system, which could be expressed as follows [39]:

(1) $$\begin{array}{c} -\nabla \cdot \left(\rho q\right)+\rho_{\text{s}}q_{\text{s}}=\rho S_{\text{f}}\frac{\partial h_{\text{f}}}{\partial t}+n\frac{\partial \rho }{\partial C}\frac{\partial C}{\partial t}, \end{array}$$

(2) $$\begin{array}{c} \frac{\partial C}{\partial t}=\nabla \cdot \left(D\cdot \nabla C\right)-\nabla \cdot \left(vC\right)-\frac{q_{\text{s}}}{n}C_{\text{s}}, \end{array}$$

where *ρ* is the fluid density, **q** is the specific discharge vector, *ρ*_s_ is the density of water entering from a source of leaving through a sink, *q*_s_ is the volumetric flow rate per unit volume of porous medium representing sources and sinks, *S*_f_ is the specific storage, *h*_f_ is the freshwater head, *n* is the porosity, *C* is the solute concentration, **D** is the hydrodynamic dispersion tensor, **v** is the fluid velocity vector, and *C*_s_ is the solute concentration of water entering or leaving through sources and sinks, respectively. Because solute reaction is not considered, fluid density is only a function of the solute concentration *C*, according to the following equation:

(3) $$\begin{array}{c} \rho =\rho_{\text{o}}\left(1+\frac{\varepsilon }{\left(C_{\text{s}}-C_{\text{o}}\right)}\left(C-C_{\text{o}}\right)\right), \end{array}$$

in which *ρ*_o_ is the freshwater density, *ε* is the density difference ratio (equation (4)), *C*_o_ is the reference concentration, and *C*_s_ is the maximum concentration. In this study, the following values are used for the parameters of equation (3): *ρ*_o_=1000 kg/m^3^, *C*_o_=0 kg/m^3^, and *C*_s_=35 kg/m^3^.

The density difference ratio is expressed as

(4) $$\begin{array}{c} \varepsilon =\frac{\rho_{\text{s}}-\rho_{\text{o}}}{\rho_{\text{o}}}, \end{array}$$

where *ρ*_s_ stands for the maximum seawater density. In this study, we consider *ρ*_s_=1025 kg/m^3^.

The Darcy flux term **q** of equation (1) for constant viscosity and freshwater properties could be expressed as

(5) $$\begin{array}{c} q_{x}=-K_{\text{f}x}\left(\frac{\partial h_{\text{f}}}{\partial x}\right), \end{array}$$

(6) $$\begin{array}{c} \;q_{y}=-K_{\text{f}y}\left(\frac{\partial h_{\text{f}}}{\partial y}\right), \end{array}$$

(7) $$\begin{array}{c} q_{z}=-K_{\text{f}z}\left(\frac{\partial h_{\text{f}}}{\partial z}+\frac{\rho -\rho_{\text{f}}}{\rho }\right), \end{array}$$

where *q*_*x*_, *q*_*y*_, and *q*_*z*_ are the components of the specific discharge in the principal directions, *K*_f*x*_, *K*_f*y*_, and *K*_f*z*_ are the components of the freshwater hydraulic conductivity in the same directions, and *ρ*_f_ is the freshwater density.

Equations (1) to (7) are the mathematical representation of the VD approach of seawater intrusion. The well-established SEAWAT code is used to solve numerically the aforementioned equation set. SEAWAT is a modular finite difference computer code created by USGS, which couples MODFLOW and MT3DMS, to solve iteratively the fluid flow and solute transport equations [39].

### 3.2. Coastal Aquifer Case Study

The VD model is applied on a rectangular-shaped unconfined aquifer. The dimensions of the aquifer model are *L*=7000 m, *W*=3000 m, and *d*=25 m. The examined aquifer geometrically resembles a real coastal aquifer located at the central eastern part of the Greek island Kalymnos, specifically the elongate aquifer underlying the Vathi valley [40, 41]. It should be noted that the examined model is an abstraction of the real aquifer, which could be considered as a typical aquifer example for the Aegean Greek islands, in terms of size and shape.

**Figure 1:**
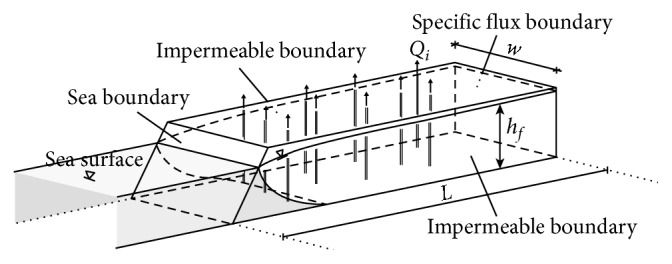
3D representation of the examined coastal aquifer.

Figure 1 outlines the conceptual model of the aquifer model. A hydrostatic boundary condition (BC) is assigned on the seaside boundary. The aquifer is bounded by impermeable geological formations, with the exception of the inland boundary, where a specified flux BC is applied to simulate the lateral inflow from the adjacent aquifer. The groundwater is replenished by a constant recharge, which is uniformly distributed along the entire surface of the aquifer. For simplification purposes, the aquifer is considered homogeneous and an anisotropic factor is assumed, which represents the differential permeability along the vertical direction.  Table 1 presents the values of the basic fluid flow and solute transport parameters. An initial simulation for approximately 200 yr without pumping was performed, until steady flow/steady transport conditions are achieved. The final hydraulic head and concentration values of this simulation are used as the initial conditions for the SI simulations, which are related to the training of the surrogate models. All simulations in the current paper are considered steady state, regarding the fluid flow conditions. This assumption resulted in relatively brief VD simulations, which allowed for the creation of an adequate sample for the calibration of the surrogate models. The duration of each simulation was approximately 1–2 min. The simulations were performed in an i7-4770 quad-core processor 3.4 GHz, with 8 GB RAM.

The 0.5 kg/m^3^ iso-chlore is considered as an indicative surface, representing the seawater intrusion wedge. Specifically, the location of the intersection of the iso-chlore and the aquifer bottom, known as the toe of the wedge, is used as a measure of the seawater intrusion extend. Further details concerning the calculation of the toe are discussed in the following sections.

## 4. Gaussian Process Regression and Other Models in Seawater Intrusion

### 4.1. Gaussian Process Regression

Gaussian process regression (GPR) is a nonparametric kernel-based probabilistic model [8]. Just like other Bayesian methods, GPR do not aim at finding “best-fit” models of the data by relating the underling function *f*(**x**) to a specific form (e.g., linear or quadratic). Instead, they calculate posterior predictive distributions for new test inputs. Such an approach enables the quantification of uncertainty as regards model estimates, as well as leveraging the understanding of the uncertainty to improve the robustness of predictions on future test points [43].

Gaussian processes can be considered as the extension of multivariate Gaussians to infinite-sized collections of variables of real value. More specifically, a Gaussian process is a collection of random variables {*f*(**x**) : **x** ∈ X} defined by its mean function *μ*(**x**) and a covariance function *k*(**x**, **x**′) so that

(8) $$\begin{array}{c} \left[\begin{array}{c} f\left(x_{1}\right)\\ \vdots \\ f\left(x_{n}\right) \end{array}\right]∼N\left(\left[\begin{array}{c} \mu \left(x_{1}\right)\;\\ \vdots \\ \mu \left(x_{n}\right)\; \end{array}\right]\left[\begin{array}{ccc} k\left(x_{1},x_{1}\right) & \ldots & k\left(x_{1},x_{n}\right)\\ \vdots & \ddots & \vdots \\ k\left(x_{n},x_{1}\right) & \ldots & k\left(x_{n},x_{n}\right) \end{array}\right]\right). \end{array}$$

The above statement can be rewritten as follows:

(9) $$\begin{array}{c} f\left(\cdot \right)∼\mathrm{GP}\left(\mu \left(\cdot \right),k\left(\cdot ,\cdot \right)\right), \end{array}$$

where each dimension of the Gaussian corresponds to an element *x* from the index set X. Furthermore, the respective component of the random vector represents the *f*(**x**) value. Typically, the prior distribution over functions *f*(·) is expected to be a zero-mean GP prior.

Consider a training set ℒ={(**x**_*i*_, *y*_*i*_)}_*i*=1_^*n*^ of i.i.d. examples from some unknown distribution, where **x**_*i*_ ∈ *ℝ*^*d*^and *y*_*i*_ ∈ *ℝ*. A GPR model assumes that a response *y*_*i*_ satisfies the following equation:

(10) $$\begin{array}{c} y_{i}=\;\;f\left(x_{i}\right)+\epsilon_{i}, \end{array}$$

where *ϵ*_*i*_ are i.i.d. noise variables, so that *ϵ* ~ *𝒩*(0, *σ*^2^). Let *𝒰*={(**x**_*i*_^(*u*)^, *y*_*i*_^(*u*)^)}_*i*=1_^*n*^ be a set of i.i.d. testing points drawn from the same unknown distribution as *ℒ*. Recall that both training and test points must have a joint multivariate Gaussian distribution.

Then, it can be proved that [1]

(11) $$\begin{array}{c} {\bar{y}}^{\left(u\right)}\left|\;\bar{y},X,X^{u}\right.∼N\left(\mu^{\left(u\right)},\Sigma^{\left(u\right)}\right), \end{array}$$

with mean value and covariance defined as

(12) $$\begin{array}{c} \mu^{\left(u\right)}=\;K\left(X^{\left(u\right)},X\right)\cdot {\left(K\left(X,X\right)+\sigma^{2}\cdot I\right)}^{-1}\cdot \bar{y}, \end{array}$$

(13) $$\begin{array}{c} \Sigma^{\left(u\right)}=K\left(X^{\left(u\right)},X^{\left(u\right)}\right)+\sigma^{2}\cdot I-K\left(X^{\left(u\right)},X\right)\cdot {\left(K\left(X,X\right)+\sigma^{2}\cdot I\right)}^{-1}\cdot K\left(X,X^{\left(u\right)}\right), \end{array}$$

respectively. Note that *K*(**X**^(*u*)^, **X**) ∈ *ℝ*^*n*×*n*^ is defined as *K*(**X**^(*u*)^, **X**)_*ij*_=*k*(**x**_*i*_^(*u*)^, **x**_*j*_), *i*, *j*=1,…, *n*. The same hold for the *K*(**X**, **X**), *K*(**X**^(*u*)^, **X**^(*u*)^), and *K*(**X**, **X**^(*u*)^) cases.

Additionally,

(14) $$\begin{array}{c} \bar{y}={\left[y_{1},\ldots ,y_{n}\right]}^{T},\\ {\bar{y}}^{\left(u\right)}={\left[y_{1}^{\left(u\right)},\ldots ,y_{n}^{\left(u\right)}\right]}^{T},\\ X=\left[\begin{array}{ccc} x_{11} & \ldots & x_{1m}\\ \vdots & \ddots & \vdots \\ x_{n1} & \ldots & x_{nm} \end{array}\right],\;\;X\in R^{n\times m}\text{ ,} \end{array}$$

**X**^(*u*)^ is defined in a similar way.

As such, we can estimate any new value as the mean of a posterior predictive distribution. We should also note that with the rise of training samples number, the confidence region size reduces, so as to reflect the decreasing uncertainty in the model estimates.

### 4.2. Alternative Regressors for Comparison

Regression trees is an alternative approach to nonlinear regression. The core idea lies in sub-dividing the space into smaller regions and then fit simple models to them [42, 44]. Provided a training set *ℒ*, a set of branches is created. Each binary split is performed according to a specific feature (from the *m* available). Then, a new value prediction is defined as

(15) $$\begin{array}{c} y\left(x\right)=\frac{1}{c}\overset{c}{\underset{i=1}{\sum }}y_{i}, \end{array}$$

where *c* is the number of observations available at the specific cell.

Support Vector Machine regression is another approach. The function used to predict new values (for linear support vector regression) is defined as [45]

(16) $$\begin{array}{c} y\left(x\right)=\overset{n}{\underset{i=1}{\sum }}\left(\alpha_{i}-\alpha_{i}^{*}\right)\cdot x_{i},x+b, \end{array}$$

where 〈·, ·〉 is the dot product, **α**_*i*_, **α**_*i*_^*∗*^ are Langrage multipliers, so that **α**_*i*_ · **α**_*i*_^*∗*^=0, *i*=1,…, *n*, and *b* is a bias term. SV algorithm can be made nonlinear by simply preprocessing the training patterns **x**_*i*_ using a kernel function *k*(·, ·). The regression is performed as

(17) $$\begin{array}{c} y\left(x\right)=\overset{n}{\underset{i=1}{\sum }}\left(\alpha_{i}-\alpha_{i}^{*}\right)\cdot k\left(x_{i},x\right)+b. \end{array}$$

### 4.3. Bayesian Optimization of Model Parameters

A variety of widely used machine learning techniques contain a significant number of parameters to be decided (e.g., SVM kernel type and parameters and ANN layers and type of activation functions). The performance of any algorithm depends on the selection of these hyperparameters [46–48]. Typically, hyperparameter tuning involves grid search, random search, and genetic algorithms, among many other techniques [49]. Such techniques require many (nonconvex) function evaluations.

Bayesian optimization (BayesOpt) is a surrogate modelling technique that can optimize an objective function that is expensive to evaluate, reducing the number of actual function evaluations required [50, 51]. It is built on Bayesian inference and Gaussian processes and is applicable in cases where closed-form expression for the objective function is not known but can obtain observations (possibly noisy) of this function at sampled values.

BayesOpt builds a probabilistic proxy model for the objective, using outcomes of past experiments as training data. The proxy model (e.g., Gaussian process) is much cheaper to calculate but it can provide adequate information on where we should evaluate the true objective function to get a good result. Assume a vector **P**={**p**_1_,…, **p**_*m*_} for a set of *m* hyperparameters to be tuned. Given a set of training paradigms {(**x**_*i*_, *y*_*i*_)}_*i*=1_^*n*^, we need to find $P^{*}=\underset{P}{\text{argmin}}g\left(P\left|{\left\{\left(x_{i},y_{i}\right)\right\}}_{i=1}^{n}\right.\right)$, where *g* is a cost function (e.g., cross-entropy cost and quadratic cost).

The entire optimization approach is guided by an appropriate acquisition function (AF), which defines the next point (i.e., set of hyperparameters) to be evaluated. As such, any AF needs to balance between exploration and exploitation.

Exploration refers to region search where the uncertainty is high, expecting to find a new set of parameters that improve model's performance. Exploitation, on the other hand, is a region search close to already calculated high estimated values (i.e., regression performance scores).

## 5. Experimental Evaluation

### 5.1. Data Preprocessing and Experiment Setup

The training sample consists of 4000 variable sets. Each set has 40 input variables: (1) the pumping rates of the 10 wells and (2) the distance of the SI toe from 30 observation points, uniformly distributed across the sea boundary. The Latin hypercube sample (LHS) statistical method was used to generate the 4000 pumping rate patterns. As mentioned in Section 2.2, the 0.5 kg/m^3^ is selected as an indicative concentration value for the SI extend. The distance between the toe and the observation wells represents the initial position of the SI wedge. Regarding the initial position of the SI toe, the variable sets are divided into four categories of 1,000 samples. In the first category, the concentration results from the zero-pumping rate simulation define the initial location of the SI toe. In the remaining categories, the solute transport and hydraulic head results of the previous category simulations are used as the initial conditions for the following simulations.

**Figure 2:**
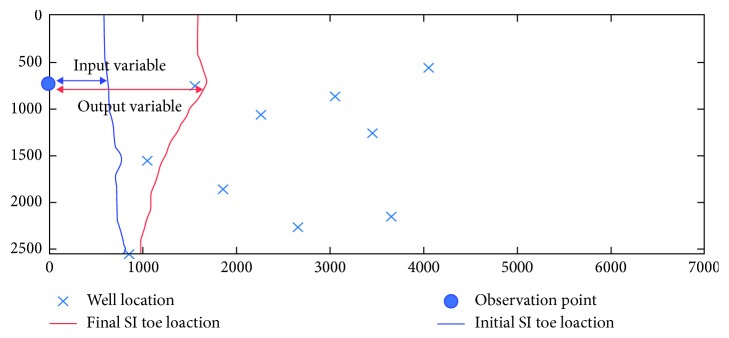
Initial and final position of the SI toe.

The output set consists of 30 variables, which represent the final location of the SI toe, calculated as the distance from the same observation points.  Figure 2 presents the initial and final position of the SI toe for a specific set of pumping rates, representing, along with the pumping rates, the input/output variables used to train the surrogate model.

### 5.2. Experimental Results

#### 5.2.1. Hyperparameter Optimization

Each regressor's hyperparameters were optimized using Bayesian optimization over 5k-fold cross-validation sets. The final parameter values are summarized in Table 2. An interesting remark is that, for the specific setup, simpler models (i.e., least square regression vs linear kernel SVMs, and linear kernel SVM vs Gaussian RBF or polynomial kernels) perform slightly better, during the optimization process.

**Figure 3:**
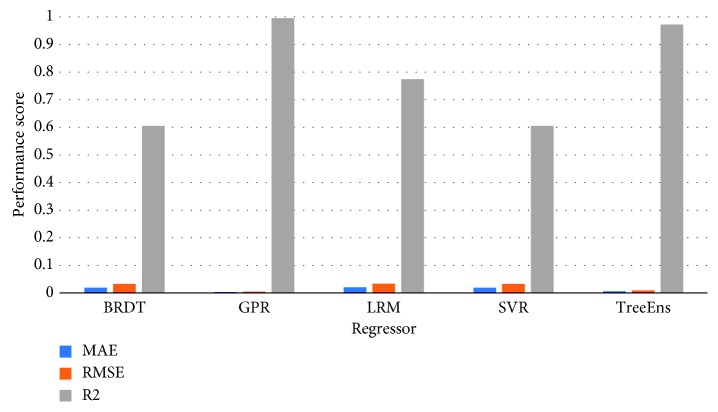
Normalized performance scores for the training set.

**Figure 4:**
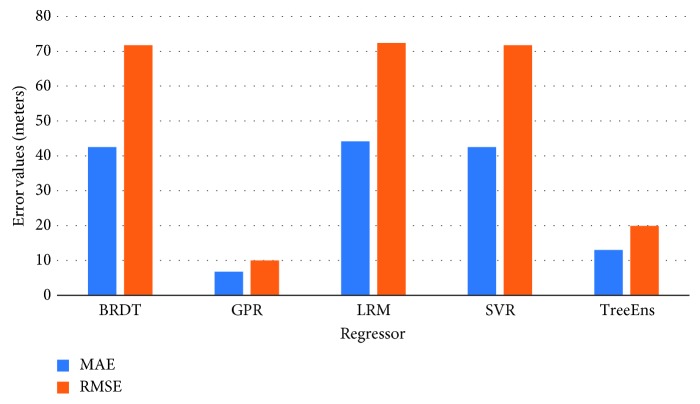
Error values (meters) for the training set.

Figure 3 illustrates the normalized performance scores of the investigated regressors for the training set, and Figure 4 provides a further insight into the actual differences (in meters), on average, for the trained models. Errors in estimation do not surpass 10 meters for the GPR and 20 meters for the TreeEns. The other regressors achieve an average error greater than 40 meters.

**Figure 5:**
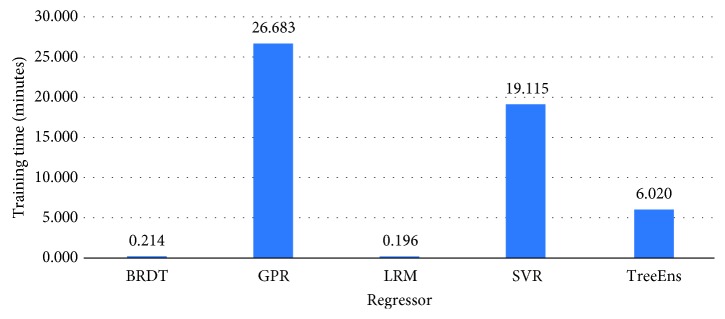
Optimization time for the regressors' hyperparameter estimation.

**Figure 6:**
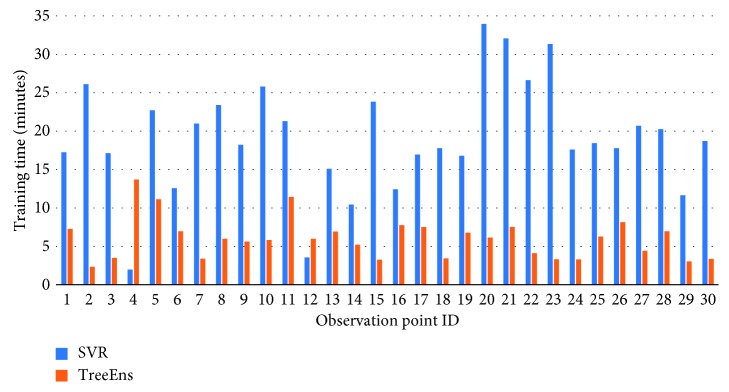
Optimization time variance, depending on the observation point data used for training.

Figure 5 illustrates the optimization time (in minutes) required for the identification of the best possible hyperparameters, using Bayesian optimization. GPR and SVR had significantly higher training times. It is also intriguing that, for different observation points, SVR and TreeEns regressors' optimization times had increased variance.  Figure 6 illustrates the case.

#### 5.2.2. Statistical Evaluation

Statistical errors calculate the sum of differences between actual (simulated) and forecasted (regressor estimated) values. The statistical measurements used were the mean absolute error (MAE) and the root mean square error (RMSE). Low error scores suggest a good regression model.

An additional performance score, i.e., coefficient of determination, *R*^2^, is used. *R*^2^ provides a measure of how well-observed outcomes are replicated by the model, based on the proportion of total variation of outcomes explained by the model. Values close to 1, i.e., *R*^2^ ≈ 1, indicate that the regression predictions approximate extremely well the actual data outputs.

**Figure 7:**
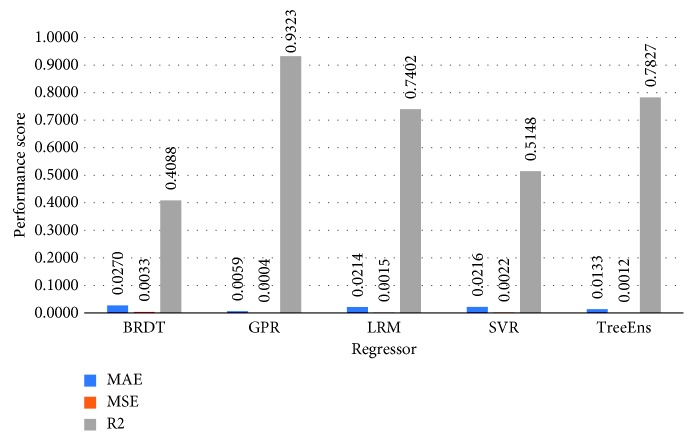
Average performance scores for each of the proposed regressors. GPR performs all other approaches in both error scores MAE and RMSE (lower the better) and coefficient of determination values (higher the better).

Figure 7 illustrates the average performance scores, for the proposed statistical errors. GPR surpasses all other repressors in all performance fields.

#### 5.2.3. Measuring Actual SI Toe Location Estimation Error

**Figure 8:**
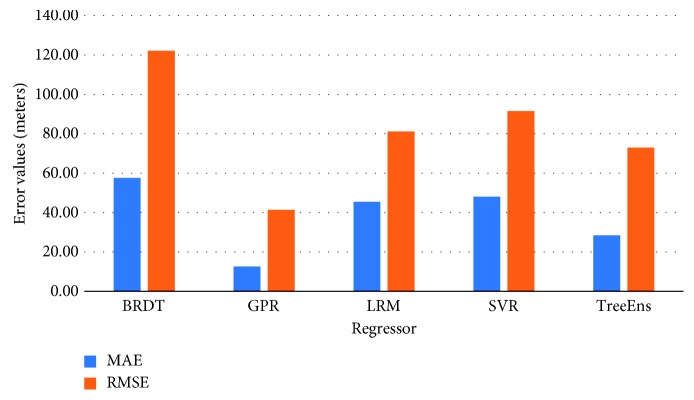
Discrepancy between actual and estimated distances for all observation points and regressors.

The statistical errors provide various information regarding the model performance. However, in our case, actual errors in the SI toe location estimations, measured in millimeters (mm), provide a deeper understanding of the regressors' performance.  Figure 8 presents the discrepancies between the actual and the estimated location of the SI toe, on average, for the five examined regressors. The comparative results indicate that the GPR method is overall more efficient and achieves more accurate prediction of the SI.

However, average scores fail to indicate the performance for each of the observation points, using each of the proposed regressors.  Table 3 provides a further insight into the error values (meters) for each of the observation points.

#### 5.2.4. Analysis of Variance

To obtain further insights into the results and the relative performance of the different algorithms, we conducted an analysis of variance (ANOVA) on the distance between the SI toe and the observation points score results for the test samples. The MAE score, in meters, represents a significant amount of information about the overall performance. Using this method, we can study the effects that the main design factors have [52].

Table 4 displays the outcomes of ANOVA. In this table, the “Source” column corresponds to the source of variation in data (i.e., the regressors and the observation points). Sum and mean sq. correspond to mean measurements between the *m* groups and the grand mean; it is a means of quantification of the variability among the groups of interest. For the degrees of freedom (d.f.), it holds that d.f.=*m* − 1. The *F* metric corresponds to the “average” intergroup variability divided by the “average” intragroup variability. The last column includes the *p* value, which is derived by comparing the *F*-statistic to an *F*-distribution with *m* − 1 numerator degrees of freedom and *n* − *m* denominator degrees of freedom, for the total set of *n* observations.

As can be seen in Table 4, both regressors and observation points have a crucial role in explaining variations in RMSE score, given the fact that the respective *p* value is approximately zero. The Tukey's honest significant difference (HSD) post hoc test is also employed to identify sampling schemes and classifiers that provide the best results, while taking into consideration the statistical significance of the differences between the results.

**Figure 9:**
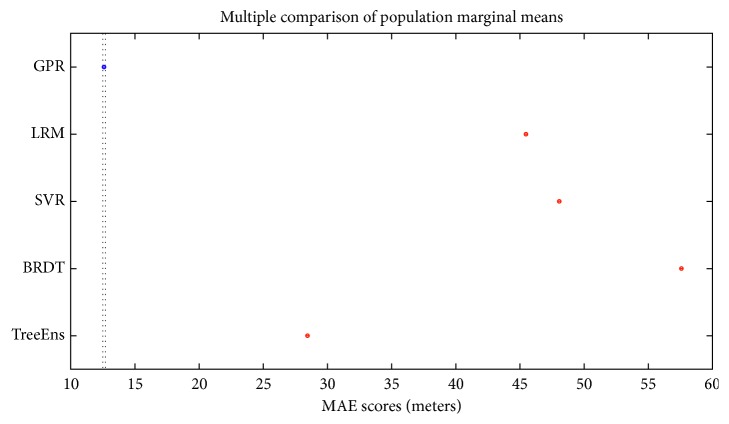
MAE scores for different types of regressors.

Figure 9 indicates that GPR by far surpasses the other regressors' MAE score. Mean scores for each regressor are shown as ‘o'. The average scores from the subgroups in the experiment are also provided, in the form of a horizontal line. Because there is no overlap between the RMSE values for the GPR type compared with the other regressors, GPR scores are clearly statistically better than the others [53, 54].

**Figure 10:**
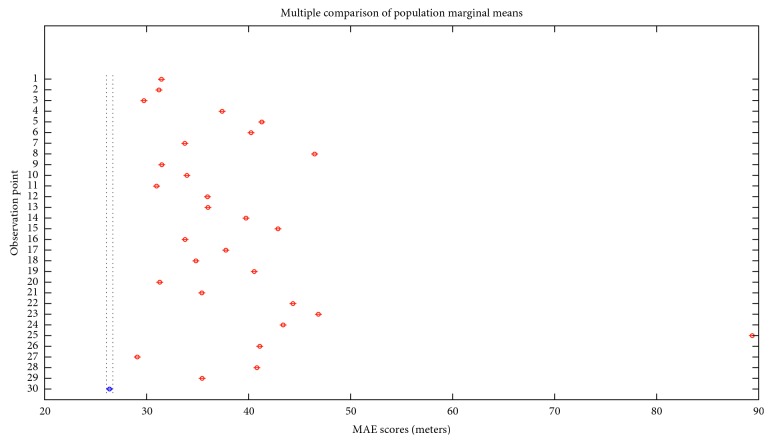
MAE scores for different observation points' positions.

Figure 10 indicates that the best observation point is point no. 30. A slight overlap in MAE subgroups' scores with point 27 (2^nd^ best observation point) is observed.

## 6. Conclusion

The present study performs a comparative analysis of four different surrogate models for the variable density approach of seawater intrusion, in particular Gaussian process regression, binary regression decision tree method, ensemble tree learners, and the support vector machine regression models. Emphasis was given on the optimization of the examined techniques. To this end, a Bayesian optimization procedure of the surrogate models hyperparameters is used. The evaluation results indicate that the GPR method surpasses the other regressors in terms of the mean absolute error (MAE), the root mean square error (RMSE), and the coefficient of determination (*R*^2^). It should be noted that GPR is significantly more time consuming. In summary, the GPR method is a reliable and accurate surrogate model for SI and could be incorporated in a pumping optimization framework in coastal aquifers. Future research will focus on further scrutinizing the effectiveness of the GPR method for saltwater intrusion prediction, compared with other well-established surrogate models.

## Figures and Tables

**Table 1:** Basic parameters of the flow and transport model (source [42]).

Parameters	Values
*K*_*x*_	100 m/d
*K*_*y*_	100 m/d
*K*_*z*_	1 m/d
Longitudinal dispersivity	50 m
Transverse dispersivity	5 m
Vertical dispersivity	0.5 m
Density ratio	0.025
Recharge	8.22 × 10^−5^ m/d
Lateral inflow	3696 m^3^/d

**Table 2:** Value ranges of optimized hyperparameters of regressors.

Regressor name	Parameter(s) name(s)	Observed value range (number of points appeared)
GPR	Kernel function	Squared exponential (30/30)
	Sigma	0.013 ± 0.005 (26/30)

LRM	Learner	Least squares
	Initial bias	−0.85 ± 0.04 (30/30)

SVM	Kernel function	Polynomial (8/20)
		Gaussian RBF (11/30)
		Linear (11/30)

BRDT	Max splits	500 ± 200 (8/30)
		900 ± 200 (8/30)
		1300 ± 200 (3/30)
		≥1501 (11/30)
	Number of variables to sample	All

TreeEns	Number of learners	200 ± 200 (17/30)
		≥400 (13/30)

**Table 3:** Detailed error values (in meters) per observation point.

	BRDT		GPR		LRM		SVR		TreeEns	
Point ID	MAE	RMSE	MAE	RMSE	MAE	RMSE	MAE	RMSE	MAE	RMSE
1	58.73	124.27	10.40	45.88	41.80	82.70	14.43	76.07	31.93	80.75
2	57.45	110.07	11.10	47.58	41.46	81.62	16.58	55.14	29.48	95.61
3	59.78	132.30	12.46	52.10	42.30	84.68	12.02	52.18	22.15	49.13
4	58.55	127.25	14.78	48.58	46.34	82.30	41.16	90.72	26.19	82.46
5	65.54	135.78	19.10	57.30	51.83	87.72	23.05	56.86	46.93	89.04
6	67.87	146.06	15.01	54.75	47.48	91.85	41.46	101.26	29.38	102.24
7	62.90	125.48	13.70	51.09	44.34	95.78	24.12	98.33	23.69	81.39
8	60.40	130.50	10.43	47.40	42.82	84.43	91.60	127.27	27.11	54.66
9	62.58	136.19	13.33	44.25	43.71	84.97	12.60	53.97	25.24	69.97
10	55.96	118.68	14.24	41.08	44.15	78.52	30.35	42.57	25.07	84.73
11	59.04	124.85	10.21	44.13	46.02	82.87	14.18	52.86	25.44	83.98
12	56.08	112.88	10.95	43.10	46.64	81.33	38.15	86.49	28.04	56.87
13	59.74	113.97	11.75	39.19	47.93	77.56	36.76	90.79	23.91	71.20
14	63.33	126.04	14.21	45.23	52.04	82.21	38.14	90.45	31.00	72.95
15	61.31	121.07	21.54	45.34	56.52	83.13	43.26	94.37	31.78	57.03
16	63.08	126.62	12.80	36.78	51.33	78.68	15.42	43.84	26.24	59.00
17	61.16	122.98	16.78	38.38	48.91	78.25	37.91	86.90	24.08	66.75
18	61.55	124.98	10.25	34.19	47.96	77.53	31.53	56.39	22.88	51.39
19	56.87	126.95	10.47	33.29	47.13	81.79	48.32	82.92	39.96	70.75
20	55.55	117.58	11.32	35.46	47.19	84.37	15.19	42.14	27.20	85.57
21	56.78	129.45	11.99	37.63	46.57	82.85	32.17	96.65	29.58	68.91
22	58.32	134.78	13.21	40.35	46.35	82.16	68.38	86.52	35.45	75.93
23	54.20	116.94	12.55	38.86	45.98	83.04	89.78	153.93	31.69	90.52
24	54.40	118.41	12.00	35.88	43.58	77.67	71.91	149.64	35.01	77.75
25	51.40	115.55	10.96	33.83	41.34	75.98	311.01	314.78	32.07	83.58
26	50.83	109.44	10.92	35.07	40.83	74.10	81.77	139.24	21.12	58.01
27	47.07	105.99	10.59	33.14	40.99	75.23	13.93	40.47	32.87	68.18
28	48.54	109.17	10.39	33.64	40.74	75.62	81.09	139.40	23.28	65.71
29	49.81	113.71	9.85	31.42	39.57	72.40	53.63	77.97	24.38	71.77
30	48.81	109.37	10.30	36.14	40.33	73.53	12.15	65.30	20.26	64.56

**Table 4:** ANOVA outcomes.

Source	Sum sq.	d.f.	Mean sq.	*F*	*p* Value
‘Regressor'	306492.5	4	76623.113	122442.294	0.00
‘ObservationPoint'	140395.9	29	4841.237	7736.206	0.00
‘HoldoutSet'	33.7	7	4.814	7.693	0.00
‘Regressor ∗ ObservationPoint'	591604.8	116	5100.041	8149.770	0.00
‘Regressor ∗ HoldoutSet'	16.8	28	0.599	0.958	0.52
‘ObservationPoint ∗ HoldoutSet'	1297.4	203	6.391	10.213	0.00
‘Error'	508.1	812	0.626		
‘Total'	1040349.2	1199			

## Data Availability

Data are not publicly available at this point because of intellectual property restrictions, but they can be provided to anyone interested on request. The data used to support the findings of this study are available from the corresponding author upon request.
